# Improving adherence to web-based cessation programs: a randomized controlled trial study protocol

**DOI:** 10.1186/1745-6215-14-48

**Published:** 2013-02-17

**Authors:** Amanda L Graham, Sarah Cha, George D Papandonatos, Nathan K Cobb, Aaron Mushro, Ye Fang, Raymond S Niaura, David B Abrams

**Affiliations:** 1 Schroeder Institute for Tobacco Research and Policy Studies, Legacy, 1724 Massachusetts Avenue, NW Washington, DC 20036, USA; 2 Department of Oncology, Georgetown University Medical Center / Cancer Prevention and Control Program, Lombardi Comprehensive Cancer Center, Washington, DC 20007, USA; 3 Center for Statistical Sciences, Brown University, Providence, RI, 02912, USA; 4 Department of Health, Behavior and Society, The Johns Hopkins Bloomberg School of Public Health, Baltimore, MD, 21205, USA; 5 Marketing Department, Legacy, 1724 Massachusetts Avenue, NW, Washington, DC, 20036, USA

**Keywords:** Smoking cessation, Internet, Adherence, Social networks, Nicotine replacement therapy

## Abstract

**Background:**

Reducing smoking prevalence is a public health priority that can save more lives and money than almost any other known preventive intervention. Internet interventions have the potential for enormous public health impact given their broad reach and effectiveness. However, most users engage only minimally with even the best designed websites, diminishing their impact due to an insufficient ‘dose’. Two approaches to improve adherence to Internet cessation programs are integrating smokers into an online social network and providing free nicotine replacement therapy (NRT). Active participation in online communities is associated with higher rates of cessation. Integrating smokers into an online social network can increase support and may also increase utilization of cessation tools and NRT. Removing barriers to NRT may increase uptake and adherence, and may also increase use of online cessation tools as smokers look for information and support while quitting. The combination of both strategies may exert the most powerful effects on adherence compared to either strategy alone.

**Methods/Design:**

This study compares the efficacy of a smoking cessation website (WEB) alone and in conjunction with free NRT and a social network (SN) protocol designed to integrate participants into the online community. Using a 2 (SN, no SN) x 2 (NRT, no NRT) randomized, controlled factorial design with repeated measures at baseline, 3 months, and 9 months, this study will recruit N = 4,000 new members of an internet cessation program and randomize them to: 1) WEB, 2) WEB + SN, 3) WEB + NRT, or 4) WEB + SN + NRT. Hypotheses are that all interventions will outperform WEB and that WEB + SN + NRT will outperform WEB + NRT and WEB + SN on 30-day point prevalence abstinence at 9 months. Exploratory analyses will examine theory-driven hypotheses about the mediators and moderators of outcome.

**Discussion:**

Addressing adherence in internet cessation programs is critical and timely to leverage their potential public health impact. This study is innovative in its use of a social network approach to improve behavioral and pharmacological treatment utilization to improve cessation. This approach is significant for reducing tobacco’s devastating disease burden and for optimizing behavior change in other arenas where adherence is just as critical.

**Trial registration:**

ISRCTN:ISRCTN45127327

## Background

Tobacco use is the leading cause of preventable death in the United States, causing 443,000 premature deaths among adults and nearly $200 billion in total economic burden each year [1]. Currently, 20.4% of adults smoke, and reductions in smoking prevalence have stalled in recent years [2]. Accelerating the reduction in the population prevalence of smoking will require innovative approaches to reach and treat current smokers [3-5].

The internet is a promising delivery channel for tobacco cessation interventions that has the potential for enormous public health impact (reach x efficacy; [6,7]). The majority of U.S. adults are internet users [8], and internet use continues to expand through all segments of the U.S. population [9], especially among rural populations, racial/ethnic minorities, and lower income groups that are at disproportionate risk for smoking. Recent reports indicate 6 to 9% of all internet users in the United States - more than 10 million adults - search for assistance in quitting smoking each year [10,11]. The internet is well-suited to deliver the core elements of evidence-based cessation treatment [12]: 1) Practical counseling (problem solving/skills training) information and feedback can be tailored and produced immediately; 2) Social support from peers and experts can be provided in online communities; and 3) Information about pharmacotherapy can be provided, with tailored guidance for medication selection and dosing. Internet treatment can be accessed 24/7 - particularly at relapse-sensitive times - and used as long as a smoker desires. The costs of internet programs are relatively fixed, resulting in greater efficiency as use increases. Studies have shown quit rates of 7 to 26% at 6 months [13-19] and recent meta-analyses support its effectiveness [20,21]. A ‘dose response’ relationship between intensity of use (for example, number of visits, duration of use) and higher abstinence rates has also been demonstrated [13,14,22-26]. The internet is not a panacea for reaching or treating all smokers, but its current use among millions of smokers provides an extraordinary opportunity to increase population cessation rates.

Despite this enormous potential, however, the impact of internet cessation interventions is yet to be realized given the challenges of poor adherence. Adherence is traditionally defined as ‘the extent to which a person’s behaviour corresponds with agreed recommendations from a health care provider’ [27]. Since internet interventions have no specified prescriptions for use, adherence in this context may best be defined as ‘the extent to which individuals experience the content of the intervention’ [28] or more simply ‘use of the eHealth intervention over time’ [29]. Adherence is measured by website utilization metrics which may include the number of logins a participant makes during a specific period of time, the number of days between registration and last login as a measure of duration of use, the number of interactive features used, and the number of modules or materials read [29,30]. A consistent finding - across internet cessation programs and study populations - is that most users engage only minimally with even the most popular, field-tested websites [22,24,31-33]. Most users visit only one to two times and do not use many of the interactive tools, community support, or pharmacotherapy that can promote abstinence. Also referred to as engagement [16,34-38], persistence [39], non-usage attrition [40,41], and exposure [42,43] in a growing number of eHealth research studies, the problem of adherence has been observed across internet studies [28,41,44]. Low levels of adherence in internet programs is not a phenomenon unique to one or two sites, but rather an aspect of this treatment modality that has yet to be understood or fully addressed [30].

The purpose of this study is to test two independent strategies - and their interaction - to improve quit rates through greater adherence to the evidence-based components of cessation treatment (practical counseling, social support, pharmacotherapy). Increasing adherence to these treatment components could result in an extraordinary increase in population rates of cessation.

### Promoting adherence to cessation treatment via social network effects

One approach to increasing adherence in internet cessation programs - and improving treatment outcomes - lies within online social networks. Data from several sources demonstrate powerful links between participation in online social networks, web-based treatment adherence, and improved outcomes [13,37,45]. For example, Cobb *et al*. [13] found that a composite measure of website utilization intensity (number of logins x duration in minutes per login) was highly correlated with use of support resources (that is, number of emails sent and received, number of people an individual sent email to and received email from), suggesting that greater website use was largely a function of involvement in the online community. Individuals who participated in the community were almost three times as likely to have quit three months later (odds ratio = 2.71) and four times more likely to be continuously abstinent for two months or more (OR = 4.08) as compared to less intensive users, even after controlling for motivation. Quitters were more likely to have posted in forums, made an online buddy, and sent or received internal email than those who did not quit. An *et al*. [45] found that weekly peer emails may have contributed to significantly higher cessation website utilization throughout a 20-week smoking cessation study with college students. More recently, Richardson *et al*. [24] reported that compared to participants who did not access the online community on BecomeAnEX.org, those who used it one time were 1.7 times more likely to be abstinent, and those who used it two or more times were 2.2 times more likely to be abstinent even after controlling for general utilization (visits) and a broad range of demographic, smoking, and psychosocial covariates. An observational study by Poirier and Cobb [37] found that participants with social ties in a web-based program to improve well-being showed higher levels of engagement as measured by website visits, opening program emails, and completing program challenges.

Unfortunately, only a small percentage of users engage in online cessation communities either actively (that is, sending messages, posting in forums) or passively (that is, reading posts by others, viewing profiles) [26,31,46]. Since social support is a key component of cessation [47-50] and participation in an online social network may yield important benefits for cessation, it is important to investigate how to increase the percentage of users who benefit from these interactions.

This study uses a social network approach - proactive, directed, and personalized outreach from within an online community - to increase the proportion of new members that participate in and become integrated into an existing online social network. The approach centers on social networking, defined as the formation of ties [51] and evidenced by reciprocal communications between members. While there are robust associations between social network factors and improved health outcomes [52,53], there are few reports of network interventions in which ties between individuals are modified or manipulated to effect behavior change [54,55]. Network interventions may work by targeting individuals based on their position within the network, or by modifying the social networking process to alter the structure of the network. Social processes such as smoking cessation are thought to exhibit a threshold [56] or complex contagion effect [57,58] where social influence increases non-linearly as additional contacts adopt (or drop) the behavior of interest. This study is based on the premise that increasing the number of ties among members of an online network may enhance a range of behavior changes (for example, repeat visits to a website, community participation, sustained NRT use, cessation), particularly in individuals who start with no connections and might otherwise form few or no connections within the network.

### Promoting adherence to cessation treatment via free NRT

A second strategy to improve adherence in the evidence-based elements of cessation treatment is to provide smokers with access to NRT free of charge. Although NRT doubles the chances of successful cessation and is a proven quitting strategy [12,59,60], the vast majority of smokers do not use NRT when quitting [61,62]. Among the small percentage of smokers that do use NRT, the majority does not follow recommended usage guidelines for duration and/or dosage [63-65]. Providing smokers with free or reduced-cost NRT has been shown to increase initial uptake (that is, trial of NRT) [66,67]. Importantly, providing smokers with free NRT may also increase utilization of the practical counseling tools and social support available in an internet cessation program. It may induce smokers to make a quit attempt [66] during which they may be more likely to make use of internet tools to plan for cessation, to assist them in setting a quit date, and to help them learn how to cope with craving and withdrawal symptoms. They may also be more likely to turn to current and former smokers in an online community for guidance through the cessation process and for information about medication dosing or side effects. There may also be reciprocal and interactive effects, with greater internet cessation program use leading to greater NRT adherence and vice versa. Several randomized trials have examined the combined efficacy of internet cessation treatment and NRT on abstinence [18,63,68,69]. No study that we are aware of has specifically addressed whether providing NRT free of charge in conjunction with internet cessation can improve cessation outcomes through greater adherence to the problem solving/skills training, pharmacological, and social support components of treatment.

### Aims

**Figure 1:**
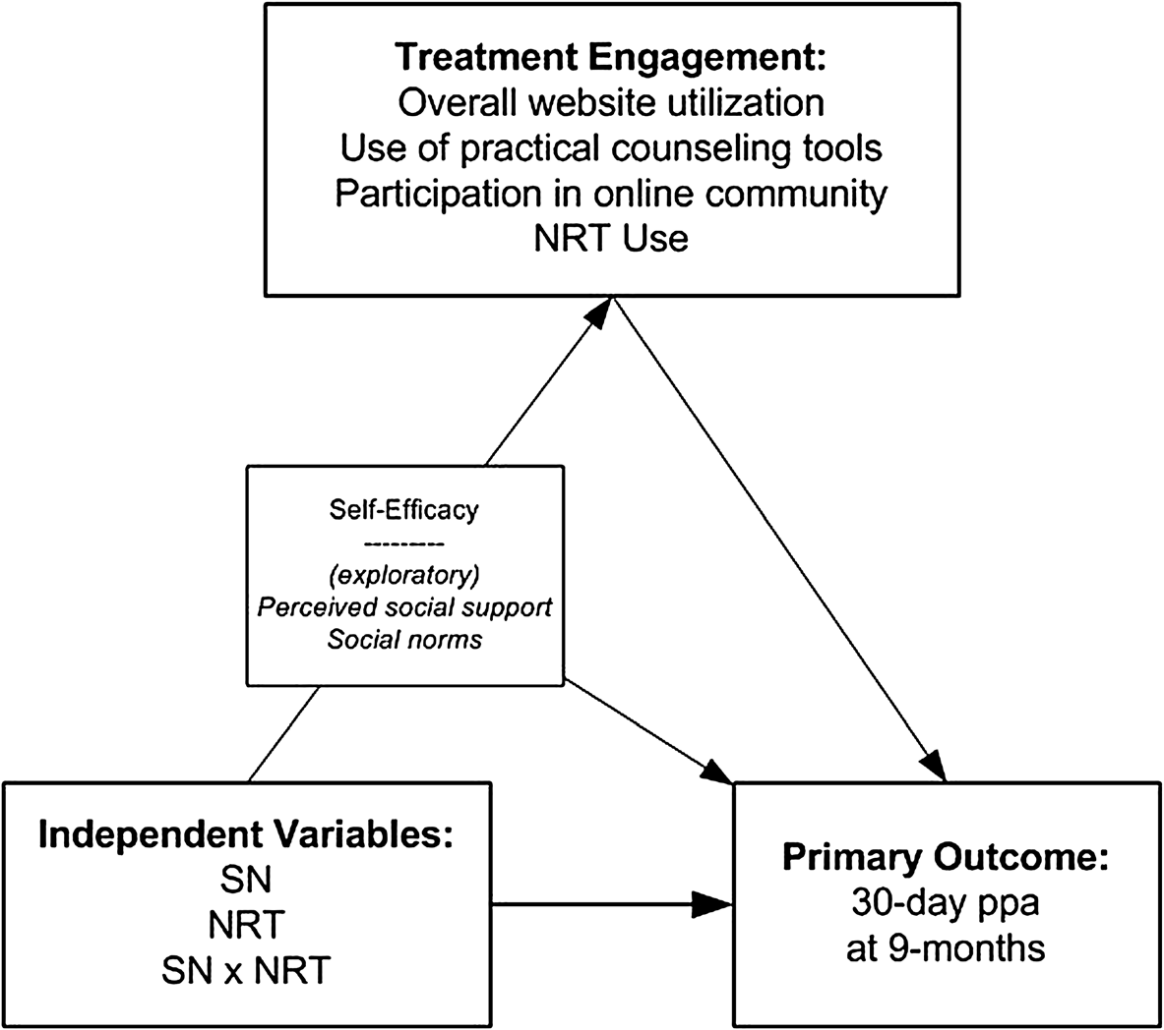
**Research model. **Theoretical model depicting the intervention components, main outcome variable, and mediating variables.

The overarching goal of this study is to test the individual and combined effects of two potentially complementary strategies to improve adherence to the evidence-based elements of cessation treatment: 1) leveraging powerful social network effects for behavior change, and 2) providing access to an initial course of free nicotine replacement therapy. Primary Aim 1 is to evaluate the comparative efficacy of WEB + SN, WEB + NRT, and WEB + SN + NRT versus WEB alone with regard to self-reported 30-day point prevalence abstinence at the primary endpoint of 9 months and at secondary endpoint of 3 months. We hypothesize that all three intervention conditions will outperform WEB and that WEB + SN + NRT will outperform WEB + SN and WEB + NRT. Primary Aim 2 is to examine whether the impact of treatment condition on cessation is mediated by greater adherence to problem solving/skills training tools, social support via the community, and pharmacotherapy (see Figure 1). We hypothesize that WEB + SN + NRT will have the greatest impact on treatment adherence, which will yield higher quit rates than the other treatments. Exploratory analyses will examine whether social support and social norms are active elements in the pathway to increased adherence, along with other known mediators of abstinence such as self-efficacy.

## Methods/Design

### Study design

**Figure 2:**
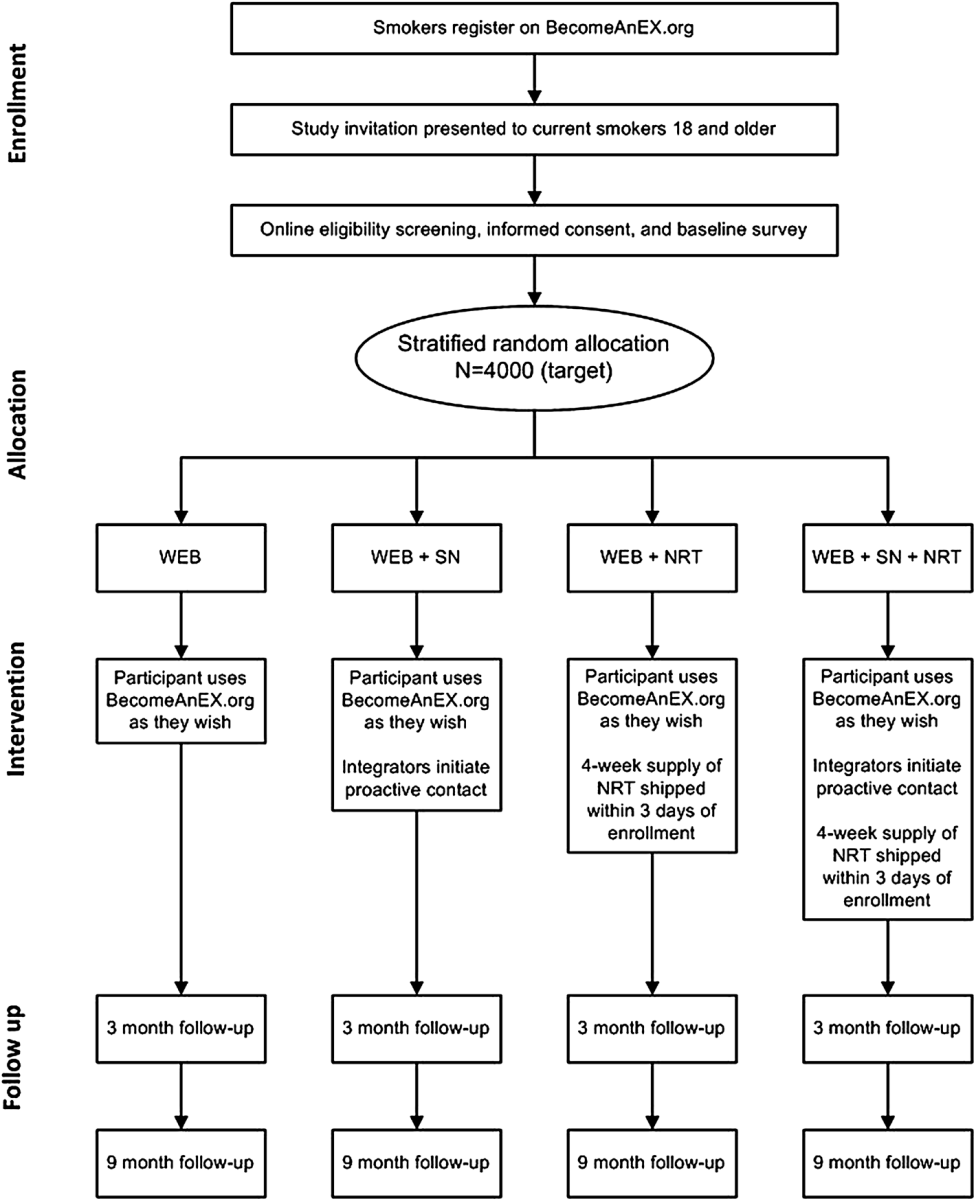
**Study procedures. **Flowchart of study procedures from enrollment through follow-up data collection.

This study is a randomized controlled trial that uses a 2 (SN, no SN) x 2 (NRT, no NRT) factorial design to compare four treatment conditions in a repeated measures design: WEB, WEB + SN, WEB + NRT, and WEB + SN + NRT. Assessments occur at baseline, at 3 months, and at 9 months, with 30-day self-reported point prevalence abstinence at 9 months as the primary outcome. Self-reported smoking status is a commonly accepted outcome measure in web-based cessation trials [14,16,18,19,22,23,70]. Biochemical verification of abstinence is not feasible on a national sample enrolled through the internet. Furthermore, misreporting of abstinence is expected to be low for several reasons: low demand characteristics of the interventions, the use of a proactive recruitment strategy to recruit a representative sample of smokers that do not have special consideration that might elicit misreporting, the fact that participation is completely under the control of the participants, and the use of an extended, 9-month follow-up period [71-74]. We will verify smoking status among a random sample of 10% of self-reported quitters with an established protocol for verification by a significant other. Study procedures from enrollment through follow-up data collection are depicted in Figure 2. The study is funded by the National Cancer Institute of the U.S. National Institutes of Health (R01 CA155489-01A1).

### Setting and participants

This is a web-based randomized trial. Participants are new registered users of BecomeAnEX.org, a free, branded, smoking cessation website developed in accordance with the Clinical Practice Guidelines [9] and publicized, in part, through a national multi-component mass media campaign that launched in 2008 [75]. Eligibility criteria are current smoking, age 18 years or older, and U.S. residence. Exclusion criteria are contraindications to nicotine replacement therapy (pregnant or breastfeeding, recent cardiac problems, current nicotine replacement therapy use).

### Recruitment

Recruitment is automated using a web-based clinical trials management system. Immediately following registration on BecomeAnEX, participants are shown an invitation that briefly describes the study. Interested participants complete an online screening form that includes questions about smoking status (current, former, never), motivation to change (planning to quit in the next 30 days, next 6 months, not planning to quit), demographic variables (gender, age, ethnicity, race, education), and contraindications to nicotine replacement therapy (Are you pregnant or breast-feeding, or do you plan to become pregnant in the next year? Do you have heart disease, an irregular heartbeat, or high blood pressure not controlled with medication, or have you had a heart attack in the past 3 months? Are you currently using any kind of stop smoking medication, such as the nicotine patch, nicotine gum, nicotine lozenge or nicotine spray, or prescription medications such as Zyban/bupropion or Chantix/varenicline?). Eligible individuals are presented with the study consent form and asked to provide online informed consent. Those who consent to participate provide contact information (name, email address, phone number). The baseline survey is sent via email to this address and must be completed within 24 hours. Participants are randomized to treatment upon completion of the baseline survey. No incentive is provided for enrollment in the study. Recruitment volume is capped at a maximum of 10 new enrollees per day to ensure a manageable workload for intervention and research staff throughout the study period.

### Randomization

Randomization is stratified by gender and baseline motivation to quit. Within-strata randomization assignments are automated using a computer algorithm.

### Interventions

#### Group 1 (WEB)

Participants have full access to the BecomeAnEX website which provides: 1) clear/strong advice to quit smoking; 2) assistance setting a quit date; 3) assessment of motivation and nicotine dependence; 4) problem-solving/skills training to enhance self-efficacy and decisional balance; 5) assistance in selecting and using FDA-approved pharmacotherapies; and 6) social support [24,75]. The voice of the site is that of a former smoker that empathizes with the challenges of quitting smoking and ‘Re-Learning’ life without cigarettes. BecomeAnEX provides practical counseling, social support, and medication information as follows:

#### Practical counseling (problem-solving/skills training)

The Re-Learn Habit section includes a series of interactive modules and videos to help users set a quit date and begin planning to quit by identifying effective/ineffective strategies from past quit attempts, removing tobacco products from their environment, identifying smoking triggers, and developing coping strategies. Extensive information is provided about how to avoid and cope with relapse. Users are provided positive coping alternatives for stress and cravings, informed about triggers for relapse, encouraged to review their reasons for quitting, and given advice on dealing with weight gain and interpersonal conflict.

#### Social support

The Re-Learn Support section emphasizes the importance of social support in the cessation process and provides guidance on how to solicit helpful support from family and friends. Members can also participate in the BecomeAnEX community. Online communications can take the form of a personal message sent directly between members, a public post on another member’s profile page (‘wall’), a discussion forum post, a forum reply to a previously posted message, or comments on a blog post. The site has over 2,000 user-initiated discussion forums and hundreds of messages are posted in the community each day. Other social support elements in the site include the ability to invite contacts from a personal email list to join the site, to search by member name within the site, and to search for and read user blogs. Clinical support blogs are posted by a tobacco treatment expert from the Mayo Clinic. Clear guidelines about appropriate community participation are provided. Administrative support for technical issues is available from the BecomeAnEX community manager and all questions are addressed within 2 business days.

#### Pharmacotherapy

The Re-Learn Addiction section provides information about nicotine addiction and FDA-approved medications. The information is presented in a series of short streaming videos narrated by a physician. The physician explains the process of nicotine addiction and addresses the myths and realities of cessation medications and how they work. Users are encouraged to use a medication in their quit attempt and to seek guidance from their pharmacist or health care provider. All seven FDA-approved cessation medications are discussed in detail.

#### Group 2 (WEB + SN)

Participants receive proactive communications from established members of the BecomeAnEX community (‘Integrators’) designed to integrate them into the community. Two former smokers who are well-known, longstanding, active members of the community serve in this role. Using a secure, dedicated interface within the study clinical trials management system, Integrators are notified when new participants have been randomized to this treatment arm. Within 24 hours, they post a public message on the new member’s ‘wall’ within BecomeAnEX to welcome them to the site, encourage them to fill out their profile, or comment on some aspect of an existing profile. In communications with participants, Integrators may use any of the following approaches to engage participants: 1) pose open-ended questions; 2) share their personal experience with smoking and quitting; 3) recommend specific tools and features of the website; 4) inquire about medication use and the participant’s experience with a chosen medication; 5) monitor the participant’s profile to determine when they have set a quit date and provide encouragement around the quit date; 6) respond to any forum posts by participants; and 7) suggest ‘friends’ or groups within the community. Integrators are blind to treatment condition in that they do not know whether participants are also receiving NRT (Group 4). The goal is for Integrators to have routine contact with participants for at least 3 months, with ongoing communication with those who respond throughout the duration of the study. Treatment duration of 3 months will provide sufficient time to examine the effectiveness of the integration protocol and will also allow us to evaluate if the effects are persistent at the 9-month follow-up. Each Integrator spends approximately 1 to 2 hours per day in this role.

Selection of the Integrators was based on analysis of key social network metrics for an online cessation community [76]. Specifically, we identified two individuals within the BecomeAnEX community who had: 1) been a member for at least 6 months; 2) logged in within the previous 2 weeks; 3) made 10 or more blog posts; 4) friended at least 25 other members; 5) both posted and received at least 500 wall comments; 6) posted at least 500 comments on blog posts; 7) joined 1 or more groups; and 8) never received any flags for offensive content. Integrators completed a one-day training to familiarize them with the aims and scope of the project, and to review recent and planned updates and modifications to the BecomeAnEX website. Unlike many other lay person/peer interventions in which those delivering the intervention are trained in the basics of tobacco dependence treatment or counseling approaches, the Integrators in this study did not receive any formal training in tobacco dependence treatment. The protocol is not designed to deliver tobacco dependence treatment or advice; rather, the overarching goal is for the Integrators to facilitate the integration of new members into the BecomeAnEX online social network.

All communications are monitored by study staff, including review of the timing, content, and tone for outgoing communications. Integrators participate in biweekly supervision meetings via teleconference.

#### Group 3 (WEB + NRT)

Participants are mailed a free 4-week supply of the NRT product of their choice (patch, gum, or lozenge) within 3 days of randomization. The decision to provide 4 weeks of free NRT (versus a full course of 8 to 12 weeks depending on product type) was based on two factors: 1) previous published studies have shown that adherence to NRT remains an issue even when participants are provided a full course of therapy [77] and that sending more free NRT to smokers does not necessarily translate into enhanced duration of use [78] or chances of quitting and remaining smoke-free [79], and 2) we are interested in examining whether providing a starter kit of free NRT increases initial uptake (that is, any use) and whether the information and resources provided on BecomeAnEX.org increase overall adherence to NRT, including purchase of additional product beyond the free supply.

Participants are notified by email following randomization that they have been assigned to a treatment group that includes NRT and again when the product has been shipped. NRT is provided as an over-the-counter product (that is, with no additional support or guidance provided) to parallel the experience subjects would have if they purchased NRT on their own. A printed calendar with study contact information is included with the NRT shipment for participants to mark the days they used the product.

#### Group 4 (WEB + SN + NRT)

Participants will have access to 4 weeks of NRT and the SN protocol as described in the sections above.

#### Additional contact

Participants in Groups 1 (WEB) and 2 (WEB + SN) are mailed a letter at the start of the study confirming that they have been enrolled. This letter is to control for the contact present in both NRT treatment arms (Groups 3 and 4) since participants in those conditions receive a mailed package at the start of the study. The content of the letter mirrors the calendar insert included in the NRT mailing.

### Data collection

Data for the study are obtained through two sources: 1) self-report assessments at baseline, at 3 months and at 9 months, and 2) online tracking software that records utilization of BecomeAnEX. Online surveys are hosted on a secure server at Legacy; all data transfer is encrypted using SSL protocols. Mixed-mode follow-up (online surveys, telephone for online non-responders; [25]) is used to maximize follow-up rates. Mailed reminders are sent prior to each follow-up. An email with a link to the online survey is sent at 3 and 9 months post-randomization; non-respondents receive reminders 3 and 6 days after the initial email. Individuals who have not responded within 7 days are contacted by telephone by professional telephone interviewers. Phone surveys are conducted by research staff blind to treatment condition. Participants are reimbursed via Amazon or PayPal for survey completion ($20 for web survey, $15 for phone survey). Individual level tracking metrics of BecomeAnEX utilization (for example, number of visits, page views, feature utilization) are recorded using Adobe/Omniture SiteCatalyst [80] software.

### Measures

Brief measures with known psychometric properties were selected when possible to minimize respondent burden. Most measures listed below are standard instruments commonly used in cessation treatment studies, and are reliable when administered via the internet [81,82] (see Table 1).

**Table 1:** Schedule of assessments

	**Baseline survey**	**Follow-up survey (3 and 9 month)**	**Omniture automated tracking**
Socio-demographic variables	X		
Internet use	X		
Smoking history	X		
Fagerström Test for Nicotine Dependence	X	X	
NRT receptivity	X		
NRT preference	X		
Behavioral intentions	X		
Personality traits	X		
**Psychosocial mediators**			
Self-efficacy	X	X	
Perceived social support	X	X	
Social norms	X	X	
**Treatment adherence mediators**			
General website utilization metrics			X
Use of practical counseling tools			X
Participation in online community			X
NRT use		X	
**Outcome measures**			
Smoking status		X	
Intervention satisfaction		X	

#### Socio-demographic variables and health status

We gather information about age, sex, marital status, race, ethnicity, employment, and education [83]. Using the item from the Medical Outcomes Study 36-Item Short-Form Health Survey (SF-36), participants rate their current health status on a 5-point scale from 1 (excellent) to 5 (poor) [84]. Participants also indicate whether they have ever had an illness caused or made worse by smoking, and whether they have been advised by a healthcare provider to quit smoking in the past year.

#### Internet use

Frequency and duration of internet use [85], type of internet connection [86], and the nature and frequency of social media use [87] are measured using survey items from the Pew Internet and American Life Project.

#### Smoking history

At baseline, current smoking and smoking history are assessed using a brief standard questionnaire which covers smoking frequency and rate; use of other tobacco products; confidence and desire to quit smoking; and quitting history including the number of quit attempts in the previous year and any methods used. Motivation to quit smoking is assessed at baseline and each follow-up using the Stages of Change algorithm [88].

#### Fagerström Test for Nicotine Dependence (FTND)

The FTND is a continuous measure of dependence and a standard instrument in the field [89].

#### NRT receptivity

Eleven questions administered at baseline address participants’ perceptions of nicotine replacement products. Items were adapted from existing instruments [90,91]. Participants rate their level of agreement with a series of statements about NRT products using a Likert scale (1 = strongly disagree; 2 = somewhat disagree; 3 = somewhat agree; 4 = strongly agree). Items include statements such as ‘NRT products double the chance of quitting compared to cold turkey’, ‘NRT products are too expensive’, and ‘The nicotine in nicotine stop smoking products is more dangerous than the nicotine in cigarettes.’

#### NRT preference

During the baseline assessment - prior to randomization - all participants indicate their preferred nicotine replacement product, selecting from a list of nicotine patch, nicotine gum, and nicotine lozenge and indicating their preferred flavor for gum and lozenge selections. Participants are reminded that not everyone will receive NRT and that selection is completely random.

#### Behavioral intentions

Sixteen items were developed to measure behavioral intentions related to the main intervention components. At baseline, participants indicate the likelihood of doing a specific action over the next 3 months using a Likert scale (1 = No, I definitely will not; 2 = I probably will not; 3 = I probably will; 4 = Yes, I definitely will). Items include statements such as ‘Keep track of my cigarettes on BecomeAnEX to identify smoking triggers,’ ‘Use BecomeAnEX regularly (that is, at least a few times a week),’ ‘Set a quit date,’ ‘Quit cold turkey without any medicine or other assistance,’ and ‘Use nicotine replacement therapy (NRT) like the patch or gum’. Psychometric analyses will be conducted to examine the factor structure and internal validity of these items.

#### Personality traits

To explore the influence of personality on adherence in a social network, participants complete six of the ten items of the Ten-Item Personality Inventory [92], a brief measure designed to assess the constellation of traits defined by the Five Factor Theory of Personality. Participants indicate the extent to which six adjective pairs describe themselves using 7-point ratings (1 = disagree strongly to 7 = agree strongly): 1) extraverted, enthusiastic, 2) reserved, quiet, 3) anxious, easily upset, 4) open to new experiences, complex, 5) calm, emotionally stable, and 6) conventional, uncreative. These six items correspond to three personality dimensions that have been linked to social media use [93]: openness to experience (items 4 and 6), extraversion (items 1 and 2), and emotional stability (items 3 and 5).

#### Self-efficacy

Smoking cessation self-efficacy is assessed using the nine-item short form of the Smoking Situations Confidence Questionnaire (SSCQ; [94]). The questionnaire can be scored to form a total situation confidence index; subscales measure confidence to resist smoking in pleasant and unpleasant situations and in situations where smoking is habitual.

#### Social support

The Appraisal and Belonging subscales of the 12-item Interpersonal Support Evaluation List (ISEL; [95]) are used to measure perceived availability of social resources at baseline. The Appraisal subscale measures the perceived availability of someone to talk to about one’s problems; the Belonging subscale assesses the perceived availability of people with whom to engage in activities. Perceptions of cessation-related support are measured at baseline and follow-up with an adapted version of the Partner Interaction Questionnaire [81,82]*.* This questionnaire assesses receipt of positive behaviors (supportive of cessation) and negative behaviors (harmful to cessation) from an individual who has followed the participant’s efforts to quit smoking. Participants are also asked how often they feel that there are people they can turn to [96] as a measure of loneliness. Response options are: 1 = Never; 2 = Rarely; 3 = Sometimes; 4 = Always. Perceptions of BecomeAnEX specific online social support is measured at each follow-up with an adapted version of the Online Social Support Scale for Smokers (OS4; [97]), a 15-item measure that assesses various dimensions of online support from a smoking cessation community.

#### Social norms

Two questions were developed to measure perceived social norms related to the two main intervention components (that is, participation in an online community, NRT use). Smokers respond to two statements: What do most smokers you know think about sharing one’s personal experiences with quitting with other people on the internet? What do most smokers you know think about nicotine replacement therapy? Response options are: 1 = They think it’s helpful for them and for most other people too; 2 = They think it’s helpful for them but not for other people; 3 = They think it’s not helpful for them but may be for other people; 4 = They think it’s not helpful for them or for anyone else.

### Outcome measures

#### Smoking status

We collect information on point prevalence smoking status at all follow-ups. The primary outcome is self-reported 30-day point prevalence abstinence measured at 9 months. Secondary outcomes include motivation to quit, number of 24-hour quit attempts, and continuous abstinence.

#### Intervention satisfaction

Participants rate their overall satisfaction, perceived helpfulness, and whether the website met their expectations on a Likert scale (1 = not at all, 5 = extremely). We also ask whether participants received any communications from other members of the BecomeAnEX community, who they received communications from, and their perceptions of those communications using ten adjectives (for example, genuine, informative, intrusive, annoying).

### Treatment adherence

#### General website utilization metrics

From BecomeAnEX we track the following general utilization metrics: number of logins, minutes spent using the site during each visit/session, number of interactive features used, and number of days logged in to the site as a measure of treatment duration. Website utilization is recorded using Adobe/Omniture SiteCatalyst. Every page view by a participant is recorded into a relational database, and page views are grouped into sessions. The duration of a session is defined as the time elapsed between the first page view and the last page view in a given session. If a user does not view a new page for more than 30 minutes, the system marks them as inactive and their next return visit creates a new session.

#### Utilization of practical counseling tools (problem solving/skills training)

From BecomeAnEX we extract data on use of specific features of the site related to practical counseling: whether the user set a quit date, number of quit date changes, use of tools to identify triggers and develop coping strategies, number of medication-related videos watched, and use of medication-related information.

#### Participation in online community

From BecomeAnEX we extract a number of metrics related to participation in the online community: completion of the social support workbook exercise, number of days between registration and community participation, number of wall posts made/received, number of forum posts/replies, number of personal messages sent/received, number of friends within the community, and extent of community profile (for example, posted photo, blog). All process data are stored in a relational database with timestamps, allowing for the extraction of specific events such as the viewing of a piece of content, the formation of a friend tie, or the content of a specific wall post.

#### Quit methods/pharmacotherapy use

At each follow-up, participants are asked about medication use and other quit methods. If participants visited a physician/health professional, they are asked whether they discussed quitting and if any cessation treatments were offered or undertaken (for example, bupropion). NRT adherence is measured at each follow-up with items that assess dosage, smoking during usage, purchase of additional NRT, and reasons for non-usage [79].

### Ethical considerations

The overall risk of this study is judged to be minimal. Participants randomized to receive nicotine replacement therapy may experience side effects. An extensive literature has generally supported the safety of nicotine patch, gum, and lozenge for healthy smokers and for distribution via population-based treatment modalities as long as clients are screened adequately per labeling instructions. This study encourages use of products that are FDA-approved and sold over-the-counter, and provides all product materials including the instructional package insert. Participants who make a quit attempt may experience withdrawal symptoms after quitting. There is no reason to believe that participation in this study would worsen nicotine withdrawal symptoms. The overall severity of withdrawal discomfort should be diminished among participants who choose to use NRT, although medication will not necessarily eliminate withdrawal discomfort entirely. In any case, withdrawal symptoms tend to be short-lived, with most symptoms abating within 1 to 2 weeks of quitting. Data security and confidentiality are protected at all times. Institutional Review Board approval for the study was provided by Western Institutional Review Board (WIRB protocol #20110887).

### Power analysis

Sample size is based on assumed quit rates of WEB = 10%, WEB + SN = 16.5%, WEB + NRT = 18%, and WEB + SN + NRT = 26% at 9 months. Assuming a non-differential attrition rate of 25% at the 9-month follow-up, these translate into Intent-To-Treat (ITT) quit rates of WEB = 7.5%, WEB + SN = 12.375%, WEB + NRT = 13.5%, and WEB + SN + NRT = 19.5%. Based upon an extensive set of simulations, we expect power to exceed 85% for all five pair-wise contrasts required for testing the hypotheses of Primary Aim 1 at a multiplicity-adjusted two-tailed significance level of 1%, chosen to maintain the study-wide Type I error rate at 5%.

Although our 2 x 2 factorial design naturally lends itself to estimation and testing of a SN x NRT interaction, interpretation of such interaction effects is highly dependent on the choice of measurement scale. For example, our assumed quit rates lead to synergistic interaction effects in the probability scale (6.5% + 8.0% for the individual SN and NRT treatments versus 16% assumed for the SN + NRT combination therapy), but antagonistic interaction effects in the odds ratio scale commonly used in logistic regression (SN versus no SN ORs that equal 1.60 when WEB + NRT is the reference group versus 1.78 when WEB alone is the reference group, leading to an SN × NRT interaction OR = 0.90). Hence, we decided to focus instead on a more clinically relevant inferential goal, that is, establishing whether the combination treatment is superior to either treatment alone, irrespective of the presence or absence of an interaction effect. However, to the extent that the presence of such a SN x NRT interaction (OR = 0.90) is of interest to the field, extensive simulation established that it can be estimated and reported with a moderate degree of precision given our planned sample size. In particular, we can be 80% certain that the length of the 95% confidence interval will not exceed 0.72 units in the OR scale (for example, 95% CI = 0.60-1.32).

### Statistical analysis plan

#### Preliminary analyses

The distributional properties of continuously scaled variables will be examined to determine the need for normalizing transformations. Next, we will determine whether the groups show large standardized mean differences at pre-treatment on demographic characteristics, psychosocial variables, or smoking variables. Although the large sample size (N = 4,000) should preclude finite sample randomization imbalances, should such between-group differences be found, we will correct for them via regression adjustment.

#### Outcome analyses

Our primary outcome for Aim 1 is self-reported 30-day point prevalence abstinence (ppa). Differences in abstinence rates between the four study conditions will be evaluated at our primary endpoint of 9 months post-randomization, as well as the secondary endpoint of 3 months post-randomization. To account for within-subject correlation due to the repeated-measures aspect of our study, we will employ the Generalized Estimating Equation (GEE), which extends generalized linear model methodology to correlated data in PROC GENMOD of SAS/STAT. Analyses will be conducted first using an ITT principle, analyzing data from all subjects randomized to treatment, counting as smokers those lost to follow-up (missing = smoking). We will also conduct a Responder-Only analysis that includes subjects reached at follow-up. Analyses will be conducted in SAS (SAS Inc., Cary, North Carolina, USA).

#### Moderator analyses

We will examine potential moderators of the intervention-smoking cessation relationship (for example, gender, baseline stage of motivational readiness, nicotine dependence). Effect modification will be conducted by analyzing interactions between treatment and selected variables.

#### Mediator analyses

**Figure 3:**
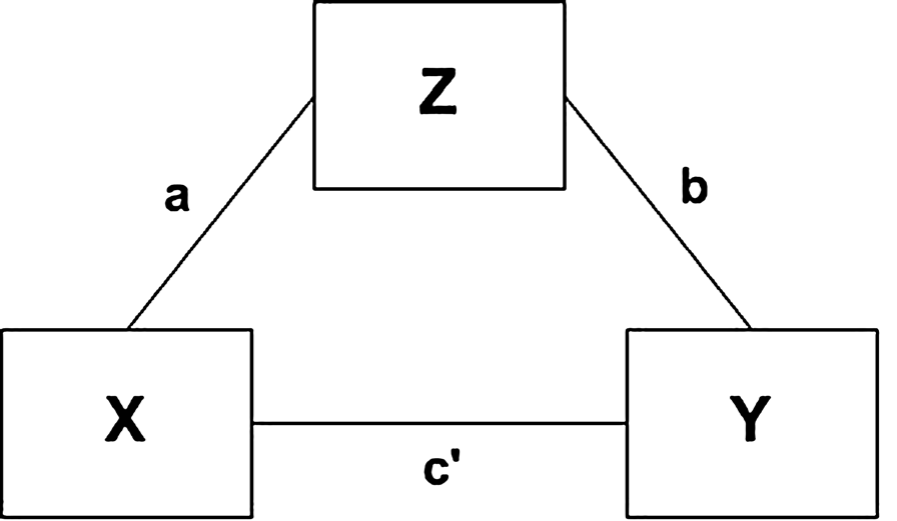
**Mediational model. **Conceptual diagram depicting the intervention (X), outcome (Y), and mediator (Z) variables as well as theory (**a**), action (**b**), and outcome (**c**’) pathways which will be examined in mediational analyses.

Primary Aim 2 hypothesizes that adherence mediates the intervention-cessation relationship. We will establish mediation using the MacKinnon approach [98]. As explained in Cerin and MacKinnon [99] and implemented by Papandonatos *et al*. [100], behavioral researchers ought to determine whether: (a) the intervention successfully acted upon the putative mediator (that is, ‘Action Theory test’); (b) changes in the mediator were indeed predictive of changes in the target behavior suggested by the conceptual framework underpinning the intervention over and above any direct treatment effects (that is, ‘Conceptual Theory test’); and (c) these conditions held simultaneously for each mediator of interest, indicating that the corresponding mediational pathway accounted for at least part of the relationship between the intervention and the target behavior (that is, ‘Mediation test’). This corresponding mediation model is shown in Figure 3. Here **X** is the exogenous variable (treatment), **Z** is the hypothesized mediator (adherence), **Y** is the outcome (abstinence or related change), **a** is the regression path between treatment and the mediator (action theory path), **b** is the regression path between the mediator and the outcome (conceptual theory path), and **c’** is the regression path between treatment and the outcome controlling for the effect of the mediator (outcome path).

#### Missing data

We expect less than 25% missing data at any time. If a participant refuses follow-up, we will censor the data at the point of loss of contact. Under an ITT approach, participants who had been considered non-smokers up to the point of loss will be considered smokers at future data points.

## Discussion

Improving adherence in internet cessation programs is critical to leverage the potential public health impact of this ‘broad reach’ treatment modality. Internet interventions are a promising delivery channel for tobacco cessation treatment that have the potential for enormous public health impact (reach x efficacy). But the impact of internet cessation interventions is yet to be realized given the limited use of effective treatment components. It is important to note that the focus of this study is not on increasing internet use among all smokers; rather, the focus is on testing specific methods to improve adherence to proven components of cessation treatment for smokers **already** using the internet to quit smoking. As internet use continues to expand through all segments of the U.S. population [9] - especially among rural populations, racial/ethnic minorities, and lower income groups at disproportionate risk for smoking - the potential of web-based interventions to reduce population prevalence of smoking will become even more important. Given the demonstrated reach of web-based interventions to millions of smokers, it is critical that we advance scientific understanding about how to engage all users so that they receive the optimal dose of treatment necessary for abstinence in order to realize the full population impact of web-based interventions. The need to improve adherence in cessation treatments is clear: even the best treatment will have little impact if it is not used.

This study is innovative in the use of a social network approach to formally manipulate and enhance social network effects (that is, individual and network level) to promote smoking cessation through improved treatment adherence. The use of dedicated staff to enhance online communities and social networks is an established industry strategy. Full time professionals typically manage a diffuse group of paraprofessionals recruited from the network itself and trained by the hosting organization [101]. This model dates back to the earliest days of online networks, such as support forums on Compuserve, and continues today where companies such as Kaiser Permanente recruit, incentivize, and train specific key-players in their online support communities to enhance discussions, recruit new members, and enforce discipline. These individuals (often called ‘peer moderators’ or ‘MVPs’) are usually managed virtually online, critically enabling the workforce to be spread across time zones. While this model is pervasive in industry, it has never been explicitly tested in a rigorous research trial with a specific focus on increasing adherence. If proven effective, this approach can be scaled to meet the demand of even the largest online health communities.

The potential scientific and public health impact of this study may extend beyond smoking cessation. Millions of adults use the internet for assistance with other health behaviors [102] and low levels of treatment utilization and attrition have been noted across eHealth studies [28,41,103]. Results from this study may inform advances in intervention design and implementation, making existing internet programs more effective across a range of behavioral risk factors. Even modest increases in the effectiveness of web-based programs can have enormous public health impact given the broad reach to millions of users.

### Limitations

We recognize that participants in Groups 1 (WEB) and 2 (WEB + SN) may use NRT on their own and participants in Groups 1 (WEB) and 3 (WEB + NRT) may participate in the BecomeAnEX social network. In an explanatory randomized trial [104] use of unassigned treatments may be considered ‘contamination’. However, in the context of a web-based pragmatic randomized trial such as this, the use of unassigned treatments is unavoidable but is expected to be minimal. That is, we anticipate that a small proportion of WEB or WEB + SN will use NRT on their own without any prompting or intervention, and a small proportion of WEB or WEB + NRT will engage in the community without any prompting or intervention. The aim of our research is to test the effectiveness of two strategies to increase the proportion of participants who adhere to all components of cessation treatment over and above the ‘usual care’ rates of use. Our power analysis, measurement instruments, and assessment protocols are designed to account for the ways in which individuals use these resources in the real-world.

Other potential limitations may be difficulty in recruiting racial/ethnic minorities in accordance with targeted enrollment projections, and difficulty in achieving follow-up rates greater than 75% as projected. In the unlikely scenario that recruitment lags, we will extend recruitment until targeted recruitment goals are met and/or modify our analytic plan accordingly. If follow-up rates are less than 75%, we will use a more intensive telephone follow-up approach and consider increasing follow-up incentives as our budget will allow.

## Trial status

Study recruitment began in March 2012. The total sample size is N = 4,000. As of January 2013, 1,700 participants have been enrolled. Recruitment is expected to be completed by summer 2014.

## Abbreviations

FDA: Food and Drug Administration; FTND: Fagerström Test for Nicotine Dependence; GEE: Generalized Estimating Equations; ITT: Intent-To-Treat; NRT: Nicotine replacement therapy; OS4: Online Social Support Scale for Smokers; PPA: Point prevalent abstinence; SN: Social network; SSCQ: Smoking Situations Confidence Questionnaire; SF-36: Medical Outcomes Study 36-Item Short-Form health Survey; ISEL: Interpersonal Support Evaluation List; OR: Odds ratio.

## Competing interests

Dr. Cobb is a consultant to MeYouHealth whose parent company's product line includes an online tobacco cessation intervention.

## Authors’ contributions

Study concept: ALG, GDP, NKC, RSN, DBA; study design: ALG, GDP, NKC, RSN, DBA; acquisition of data: ALG, SC, NKC; statistical analysis: GDP, ALG, SC, YF; draft of manuscript: ALG, SC; comments on manuscript: GDP, NKC, AM, YF, RSN, DBA; obtained funding: ALG, GDP, NKC, RSN, DBA. All authors read and approved the final manuscript.
